# A flow cytometric method for estimating S-phase duration in plants

**DOI:** 10.1093/jxb/erw367

**Published:** 2016-10-03

**Authors:** Leigh Mickelson-Young, Emily Wear, Patrick Mulvaney, Tae-Jin Lee, Eric S. Szymanski, George Allen, Linda Hanley-Bowdoin, William Thompson

**Affiliations:** ^1^Department of Plant and Microbial Biology, North Carolina State University, Raleigh, NC 27695, USA; ^2^Department of Horticultural Science, North Carolina State University, Raleigh, NC 27695, USA; ^†^ Present address: Syngenta Crop Protection, LLC, Research Triangle Park, NC 27709,USA; ^‡^ Present address: Department of Biochemistry, Duke University, Durham, NC 27710, USA

**Keywords:** Arabidopsis, barley, EdU, flow cytometry, maize, replication timing, rice, S-phase duration, wheat.

## Abstract

The duration of the DNA synthesis stage (S phase) of the cell cycle is fundamental in our understanding of cell cycle kinetics, cell proliferation, and DNA replication timing programs. Most S-phase duration estimates that exist for plants are based on indirect measurements. We present a method for directly estimating S-phase duration by pulse-labeling root tips or actively dividing suspension cells with the halogenated thymidine analog 5-ethynl-2'-deoxyuridine (EdU) and analyzing the time course of replication with bivariate flow cytometry. The transition between G_1_ and G_2_ DNA contents can be followed by measuring the mean DNA content of EdU-labeled S-phase nuclei as a function of time after the labeling pulse. We applied this technique to intact root tips of maize (*Zea mays* L.), rice (*Oryza sativa* L.), barley (*Hordeum vulgare* L.), and wheat (*Triticum aestivum* L.), and to actively dividing cell cultures of Arabidopsis (*Arabidopsis thaliana* (L.) Heynh.) and rice. Estimates of S-phase duration in root tips were remarkably consistent, varying only by ~3-fold, although the genome sizes of the species analyzed varied >40-fold.

## Highlight

We estimated S-phase duration for several plant species by following EdU-labeled nuclei from G_1_ to G_2_ using bivariate flow cytometry. S-phase duration is relatively consistent over a range of genome sizes.

## Introduction

Historically, most estimates of the duration of S phase and the cell cycle in plants have been made by analyzing the percentage of labeled mitoses (PLM) as a function of time after pulse-labeling with a DNA precursor. This technique was first described in mouse epithelium (Quastler and Sherman, 1959) and was the standard method of cell cycle analysis for nearly three decades (Van’t Hof, 1974; Grif *et al.*, 2002; Francis *et al.*, 2008, and references therein). Other methods that have been used to estimate S-phase duration in plants include double labeling (Wimber and Quastler, 1963), cell synchronization (Nagata *et al.*, 1992; Lee *et al.*, 1996; Cools *et al.*, 2010), measurement of cell doubling time (Richard *et al.*, 2001; Menges *et al.*, 2006), kinematic analysis (Dhondt *et al.*, 2010), and analysis of 5-ethynl-2'-deoxyuridine (EdU) labeling kinetics (Hayashi *et al.*, 2013; Hu *et al.*, 2015).

PLM (reviewed in Webster and Macleod, 1980; Shackney and Ritch, 1987), double labeling, and EdU labeling kinetics are limited logistically to relatively small numbers of cells. When implemented with radioactive DNA precursors (^3^H and/or ^14^C), as they were originally, PLM and double labeling methods required lengthy autoradiographic exposure times and could have direct radiological effects on the cell cycle (De La Torre and Clowes, 1974; Gould and King, 1984; Darzynkiewicz *et al.*, 1987; Fiorani and Beemster, 2006). Synchronization procedures necessarily perturb the normal cell cycle (Lee *et al.*, 1996; Dolezel *et al.*, 1999; Planchais *et al.*, 2000; Menges and Murray, 2002; Cools *et al.*, 2010). Moreover, the degree of synchronization depends on the individual characteristics of a cell population (Planchais *et al.*, 2000) and is rarely complete (Cooper, 2003), particularly in roots (Lee *et al.*, 1996). Methods based on estimates of cell doubling time are applicable mainly to isolated cells in suspension culture where, ideally, cells types are uniform and cell number and cell volume are proportional. However, a complication is the necessary assumption that all cells in suspension are cycling (Gould and King, 1984) when, in many cases, a substantial subpopulation is no longer dividing. In intact meristems, this approach is particularly complicated because meristems consist of multiple tissues and the proportionality of cell division and cell volume is generally not observed (Green, 1976; Fiorani and Beemster, 2006). Kinematic analysis requires a lengthy series of microscopic observations and complex mathematical analysis (Fiorani and Beemster, 2006; Rymen *et al.*, 2010).

These methods have yielded many novel and informative analyses of the cell cycle but, in many cases, S-phase duration was inferred from measurements of other parameters. Flow cytometry is an excellent method for detailed analysis of proliferating cell populations as it provides the distribution of nuclei based on their DNA content (Fiorani and Beemster, 2006; Darzynkiewicz *et al.*, 2011; Darzynkiewicz and Zhao, 2014). Additionally, it allows for rapid measurements of large cell populations, and the application of flow cytometry to plant systems was greatly facilitated by the development of a method for isolating and analyzing suspensions of isolated nuclei (Galbraith *et al.*, 1983). Furthermore, bivariate flow cytometric analysis has the advantage of directly measuring the increase in DNA content of a cohort of labeled (replicating) cells as they move through S phase. Bivariate flow cytometry using the halogenated DNA precursor, 5-bromo-2'-deoxyuridine (BrdU), to label cells in S phase has been successfully applied to both animal (Reviewed in Darzynkiewicz *et al.*, 2011) and plant systems (Lucretti *et al.*, 1999) and was used to elucidate the replication timing program in Arabidopsis (Lee *et al.*, 2010). When EdU is used as the DNA precursor in place of BrdU, visualization no longer requires denaturation of the DNA or cumbersome immunostaining procedures (Kotogany *et al.*, 2010). EdU labeling, which can be detected by the covalent attachment of Alexa Fluor 488 (AF 488) via Click chemistry (Rostovtsev *et al.*, 2002), has been used successfully for flow sorting and microscopic analysis of nuclei obtained from both suspension cultures and intact root tips (Bass *et al.*, 2014, 2015; Wear *et al.*, 2016).

To measure S-phase duration directly, we used a pulse–chase protocol to label DNA with EdU in a cohort of replicating cells. After various chase periods, we extracted nuclei and used bivariate flow cytometry to follow increases in the DNA content of the labeled cohort as a function of time. We estimated the average S-phase durations in the root meristems of maize (*Zea mays* L.), rice (*Oryza sativa* L.), barley (*Hordeum vulgare* L.), and wheat (*Triticum aestivum* L.), and in Arabidopsis (*Arabidopsis thaliana* (L.) Heynh.) and rice cell suspension cultures. We found that S-phase duration in grass root tips is remarkably consistent, varying by just over 3-fold in species whose genome sizes span a nearly 40-fold range. When we compare the S-phase durations of rice root tip cells with those of cultured rice cells, we found that the cultured cells take approximately twice as long to complete DNA replication.

## Materials and methods

### Plant growth

Seed of *Z. mays* L. cultivar B73 provided by Mark Milliard (GRIN NPGS, North Central Regional Plant Introduction Station, Department of Agronomy, Iowa State University, Ames, IA, USA) were increased through one generation by 27 Farms of Homestead, Inc. (Homestead, FL, USA). Seed of *O. sativa* L. cultivar Nipponbare were provided by Dr Rongda Qu (Department of Crop Science, North Carolina State University, Raleigh, NC, USA). Seed of *H. vulgare* L. cultivar Morex were provided by Dr Kevin Smith (Department of Agronomy and Plant Genetics, University of Minnesota, St. Paul, MN, USA). Seed of *T. aestivum* L. cultivar Chinese Spring were provided by Dr Gina Brown-Guidera (Department of Crop Science, USDA, North Carolina State University, Raleigh, NC, USA) and Jon Raupp (Wheat Genetics Resource Center, Department of Plant Pathology, Kansas State University, Manhattan, KS, USA). Wild-type seed of *A. thaliana* (L.) Heynh. Col-0 were provided by Mary Dallas (Department of Plant and Microbial Biology, North Carolina State University, Raleigh, NC, USA).

For grass species, 50–200 seeds were used for each time point per species. The number of seeds remained consistent among time points and biological replicates for a given species. Maize seeds were imbibed overnight in sterile, distilled water with stirring and aeration prior to surface sterilization. Maize and de-hulled rice seeds were surface sterilized in a 10% commercial bleach solution containing 0.05% Tween-20 for 15min with rotary mixing and washed 3–4 times with 2 vols of sterile water prior to germination. Twelve seeds were placed in sterile magenta boxes equipped with paper towels pre-wetted with 10ml of sterile water. Seeds were germinated under constant, fluorescent dim light (6.75 µmol photons m^−2^ s^−1^) until primary roots were 2.5–4cm long, which took 3 d for maize at 28 °C, 2 d for barley at 28 °C, 4 d for rice at 28 °C, and 3 d for wheat at 23 °C.

For Arabidopsis, 6000 seeds were used per time point. Arabidopsis seeds were surface sterilized in absolute ethanol for 5min, followed by 20% commercial bleach containing 0.05% Tween-20 for 15–20min with end-over-end mixing and washed five times with 1vol. of sterile distilled water. Seeds were stored in sterile water and vernalized in the dark at 4 °C for 72h. Three thousand seeds were germinated in rows per sterilized hydroponic dish (Alatorre-Cobos *et al.*, 2014) equipped with 300 µm mesh in 250ml of liquid Murashige and Skoog medium under constant fluorescent light (25.65 µmol photons m^−2^ s^−1^) until roots were 3–5mm long, which took 4 d at 23 °C.

### Cell cultures

An Arabidopsis (Col-0) cell line (Tanurdzic *et al.*, 2008; Lee *et al.*, 2010) was maintained in Gamborg’s B5 basal medium with minor salts (Sigma G5893) supplemented with 1.1mg l^−1^ 2,4-dichlorophenoxy acetic acid (2,4-D), 3mM MES, and 3% sucrose. Cells were grown on a rotary shaker at 160rpm under constant fluorescent light (24.3 µmol photons m^−2^ s^−1^) at 23 °C and subcultured every 7 d using a 1:8 dilution of inoculum into fresh medium.

A rice (*O. sativa* L.) cell line (Japonica cultivar Nipponbare; Lee *et al.*, 2004) was maintained in AA medium (Thompson *et al.*, 1986) supplemented with 2mg l^−1^ 2,4-D, 3mM MES, and 3% sucrose. Cells were grown on a rotary shaker at 160rpm in the dark at 27 °C and subcultured every 7 d using a 1:5 dilution of inoculum into fresh medium containing 0.005% pectinase.

### EdU labeling and fixation

**Fig. 1.**
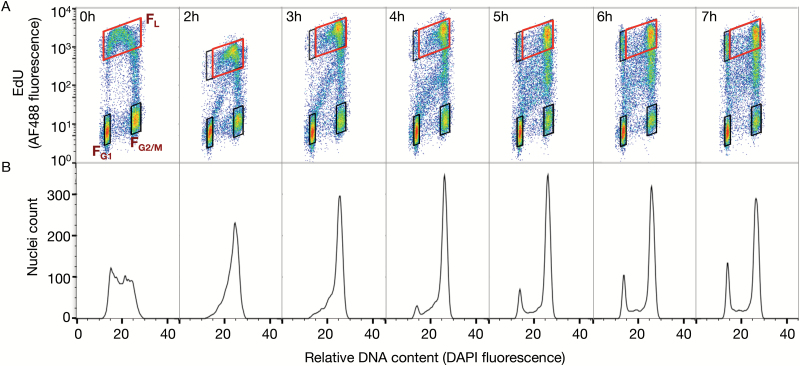
Flow cytometric analysis of an S-phase duration time course in maize root tip cells. Roots of 3-day-old maize seedlings were pulse-labeled with 25 μM EdU for 30min, washed, and then chased with 25 μM thymidine for select hourly intervals. Terminal 1mm segments of the root tips were excised, fixed, and frozen. Nuclei were prepared and analyzed by flow cytometry. (A) Bivariate flow cytograms of EdU incorporation (AF 488 fluorescence) and relative DNA content (DAPI fluorescence). The flow cytograms show labeled S-phase populations (F_L_) and unlabeled G_1_ (F_G1_) and G_2_/M (F_G2/M_) populations at various times during the chase. The mean DNA content for each population was determined by calculating the mean DAPI fluorescence within the gated region (red boxes for EdU-labeled S-phase nuclei and black boxes for unlabeled G_1_ and G_2_/M nuclei). At 0h, the gate used to calculate mean DAPI fluorescence for labeled nuclei included the entire, main S-phase arc (red box). At time points after 0h, the gate used to calculate mean DAPI fluorescence of labeled nuclei (smaller red boxes in 2–7h) excluded the labeled nuclei that had divided and returned to G_1_ (gray boxes directly above unlabeled G_1_ and juxtaposed to red boxes in flow cytograms). (B) Corresponding univariate histograms of relative DNA content (DAPI fluorescence) of EdU-labeled, S-phase nuclei. The S-phase histograms show the distribution of the main S-phase nuclei (including those that had returned to G_1_) immediately after labeling and throughout the chase and have been standardized so that each time point contains the same number of total events. The bivariate flow cytograms and corresponding histograms represent a single biological replicate.

Roots were pulse-labeled as previously described by Wear *et al.* (2016). Roots of intact seedlings were rinsed in sterile distilled water and then incubated for 30min in sterile water containing 25 μM EdU at 23 °C (wheat) or 28 °C (maize, rice, and barley) or 10 μM EdU at 23 °C (Arabidopsis roots). Incubations were carried out on a rotary shaker set to 65rpm. The roots were rinsed twice with 2–3 vols of sterile water and the EdU label was chased for various times with 25 μM (maize and rice), 100 μM (maize, rice, barley, wheat, and Arabidopsis roots), or 200 μM (wheat) thymidine prepared in sterile water. Chase conditions using varying thymidine concentrations were used to resolve the appearance of the residually labeled ‘arm’ of nuclei observed in the flow cytograms of maize, rice, and wheat (Fig. 1A; Supplementary Figs S1, S2A, C at *JXB* online and described in detail in the Results). Roots were then rinsed twice with 2–3 vols of sterile water. For grass species, terminal 1mm (maize) or 5mm (rice, barley, and wheat) root segments were excised and fixed in 1% formaldehyde in 1× phosphate-buffered saline (PBS) for 15min, with the first 5min under vacuum. The formaldehyde reaction was quenched by adding glycine to a final concentration of 0.125 M for 5min under vacuum. Fixed root tips were washed three times with 1vol. of 1× PBS, snap-frozen in liquid nitrogen, and stored at –70 °C until preparation of nuclei. For Arabidopsis roots, intact roots were fixed, quenched, and washed with the same conditions as described above and then terminal root segments ranging from 3mm to 5mm long were excised and snap-frozen as above.

Arabidopsis and rice cell suspensions were pulse-labeled as described by Wear *et al.* (2016). Four (Arabidopsis) or three (rice) days after transfer, logarithmically growing cells from a 50ml culture were labeled by adding 12.5 μl or 31.25 μl of a 40mM solution of EdU prepared in DMSO to the culture medium to a final concentration of 10 μM (Arabidopsis) or 25 μM EdU (rice), respectively. An EdU pulse concentration of 10 μM for Arabidopsis cells was used to maintain consistency with concentrations used in replication timing studies, and an EdU pulse concentration of 25 μM for rice cells was used to maintain consistency with concentrations used in labeling intact rice root meristems. Cells were incubated for 30min at 23 °C (Arabidopsis) or 27 °C (rice) on a rotary shaker set to 160rpm. The EdU was chased for various times by adding 125 μl of a 40mM solution of thymidine in sterile water to the labeled cultures to a final concentration of 100 μM. For each time point after the chase, 10ml of culture was removed and centrifuged at 400 *g* for 2min. The supernatant was removed and cells were fixed by re-suspending the pellet in 1% formaldehyde in 1× PBS and incubating at room temperature with end-over-end mixing for 15min. The formaldehyde reaction was quenched by adding glycine to a final concentration of 0.125 M and incubating at room temperature with end-over-end mixing for 5min. The fixed cells were pelleted by centrifugation at 400 *g* for 2min and the fixative/quench solution was discarded. The cell pellet was re-suspended and washed three times in 1vol. of 1× PBS with centrifugation as above between each wash step. After the final wash step, cells were pelleted by centrifugation as above and the PBS was discarded. The cell pellet was snap-frozen in liquid nitrogen and stored at –70 °C until preparation of nuclei.

### Isolation of nuclei

Nuclei were isolated by a modification of the chopping method (Galbraith *et al.*, 1983) as described by Wear *et al.* (2016). Fixed and frozen root tips or cell pellets were ground at 4 °C in 25–35ml of cell lysis buffer (CLB: 15mM Tris, pH 7.5, 2mM Na_2_EDTA, 80mM KCl, 20mM NaCl, 0.1% Triton X-100, and 15mM 2-mercaptoethanol) using the lowest setting on a commercial food processor (Cuisinart^®^ Mini-Prep^®^ Processor, model DLC-1SS). The ground suspension was incubated at 4 °C for 5min with gentle swirling half way through the incubation to maximize the yield of nuclei and then filtered through a double layer of Miracloth. The filtrate was centrifuged at 400 *g* for 5min at 4 °C and the pellet of nuclei was re-suspended in 1ml of modified CLB without EDTA and 2-mercaptoethanol.

### Click chemistry and DAPI staining

Nuclei were centrifuged at 200 *g*, 4 °C, for 5min and the supernatant was discarded. Labeled nuclei were thoroughly re-suspended and coupled to AF 488 in 0.5ml of Click-iT^®^ reaction buffer according to the manufacturer’s instructions (Click-iT^®^ EdU Alexa Fluor^®^ 488 Imaging Kit, Life Technologies). Nuclei were then centrifuged as before, washed with 2 vols of CLB, and re-suspended in 0.5–1.0ml of CLB containing 2 μg ml^−1^ DAPI. Following each centrifugation step, the nuclei were carefully and thoroughly re-suspended to minimize aggregation. The final suspension was filtered through a CellTrics 20 μm nylon filter (Partec) prior to flow cytometric analysis.

### Flow cytometric analysis

Nuclei were analyzed with an InFlux (BD Biosciences) cell analyzer/sorter equipped with a 355nm UV laser, a 488nm sapphire laser, and BD FACS™ Software v. 1.0.0.650, using 1× PBS as the sheath buffer. The software recorded forward scatter (FSC), side scatter (SSC), emission at 460/50nm (DAPI fluorescence), and emission at 530/40nm (AF 488 fluorescence). FSC was used as the trigger and to set the event threshold. Dot plots of FSC versus SSC, FSC versus DAPI fluorescence, and SSC versus DAPI fluorescence were used to locate and gate populations based on light scattering properties and DNA content (described in detail in Wear *et al.*, 2016). A bivariate dot plot of AF 488 fluorescence (log scale) versus DAPI fluorescence (linear scale) was created to visualize labeled and unlabeled nuclei at each time point in the experiment. In the plots, labeled nuclei at different stages of S phase formed an arc above the unlabeled G_1_ and G_2_ populations of nuclei.

### Relative movement

FlowJo v. 10.0.6 (Tree Star, Inc.) was used for flow cytometric data analysis. For each experiment, we analyzed at least three biological replicates conducted at different times with different batches of plant material. Within each biological replicate, we analyzed a minimum of three separate data files per time point. SSC versus DAPI plots were used to determine appropriate gates to exclude small debris from the analysis (described in detail in Wear *et al.*, 2016). AF 488 versus DAPI plots were used to define gates for labeled nuclei in S phase and unlabeled nuclei in G_1_ or G_2_/M, using similar gates for each of the biological replicates for a given species. The mean DAPI fluorescence was quantified for each of the S-phase, G_1_, and G_2_/M gated populations. At 0h (i.e. immediately after the EdU pulse-label), the gate used to calculate mean DAPI fluorescence for labeled, undivided nuclei included the entirety of the main S-phase arc (Fig. 1A, 0h, red gate). At all time points after 0h, the gate used to calculate mean DAPI fluorescence of labeled nuclei was drawn such that it excluded nuclei that had divided and returned to G_1_ (Fig. 1A, 2–7h, smaller red gates). For each time point, the relative movement (RM) of EdU-labeled nuclei in S phase was calculated by comparing their mean DAPI fluorescence relative to the mean DAPI fluorescence of the G_1_ and G_2_/M populations using the equation: RM=(F_L_–F_G1_)/(F_G2/M_–F_G1_) (Begg *et al.*, 1985), where F_L_=mean DAPI fluorescence of EdU-labeled nuclei, F_G1_=mean DAPI fluorescence of unlabeled G_1_ nuclei, and F_G2/M_=mean DAPI fluorescence of unlabeled G_2_/M nuclei. For each species, the average RM values from multiple biological replicates were combined and plotted as a function of time.

### S-phase duration

S-phase duration estimates were calculated from statistical analyses of an RM plot of combined biological replicates using R v. 3.2.3 loaded with the package ‘Segmented’ v. 0.5–1.4 (Muggeo, 2008). A generalized linear model was first applied to the data points, and the Davies test was used to test for a non-constant slope parameter. ‘Segmented’ was used to estimate a regression model with a piecewise linear relationship having a single breakpoint and two lines. The 95% confidence intervals were computed for the slope of each of the two fitted regression lines and an *R* ^2^ value was calculated. Lower and upper estimates of S-phase duration were derived from the estimated breakpoint and extrapolation of the first component line (line below the estimated breakpoint) to a theoretical RM value of 1.0.

## Results

### Flow cytometric analysis of EdU-labeled S-phase nuclei

Seedlings or cell suspension cultures were pulse-labeled with EdU and then chased with excess thymidine for various periods of time as described in the Materials and methods. At each time point, root segments or cells were fixed, nuclei were isolated, the incorporated EdU was coupled to AF 488, and total DNA was stained with DAPI. We analyzed the nuclei by flow cytometry to produce bivariate plots of relative DNA content (DAPI fluorescence) and EdU incorporation (AF 488 fluorescence) as well as univariate plots showing the distribution of relative DNA contents in the cohort of EdU-labeled (replicating) nuclei. Examples of such plots for nuclei from maize root tips are shown in Fig. 1A, B.

Three distinct populations of nuclei were identified in the bivariate plots—unlabeled nuclei in G_1_ with 2C DNA content, unlabeled nuclei in G_2_/M with 4C DNA content, and EdU-labeled S-phase nuclei with DNA contents between 2C and 4C. At the end of the labeling period (Fig. 1A, 0h), the DNA content of S-phase nuclei was intermediate between the two extremes and formed a distinct arc above the unlabeled populations in the flow cytograms. Samples harvested at various times during the thymidine chase were used to track the movement of EdU-labeled nuclei as they progressed through S phase by following increases in DNA content of the nuclei in the red box (Fig. 1A). A subset of labeled S-phase nuclei that were probably near the end of S phase when the pulse label began, divided and re-entered G_1_, creating a new group of labeled nuclei with 2C DNA content (Fig. 1A; 4–7h, gray boxes directly above unlabeled G_1_). The distribution of the main EdU-labeled nuclei population was visualized in histograms for each time point (Fig. 1B, red box at 0h and gray box plus red box at 2–7h) and confirmed the even distribution of DNA content between 2C and 4C at the end of the labeling period (Fig. 1B, 0h), the movement of the main cohort of labeled nuclei (Fig. 1B, after 0h), and the return of labeled nuclei with 2C DNA content (Fig. 1B, 4–7h).

An ‘arm’ of less intensively labeled nuclei (Fig. 1A, 2–4h) was observed below the S-phase gate and extended diagonally upwards from 2C across to 4C. These nuclei progressed towards 4C but remained below the fully labeled gated 4C nuclei, eventually forming a downward extension of the S-phase arc (Fig. 1A, 5–7h). Similar ‘arms’ were observed in the flow cytograms of rice and wheat roots (see Supplementary Fig. S2A, C at *JXB* online) and Arabidopsis cell culture (Fig. 4A). The arms most probably reflect a low level of EdU labeling that continued after the start of the chase period. In cell cultures, the chase was initiated by adding excess thymidine, without washing away the EdU. In the case of root tips, after the pulse-label was complete, excess EdU was washed away from the root tips before placing them in the thymidine solution. However, it is likely that some EdU remained in intercellular spaces and intracellular pools. In both cases, it may have taken some time for the thymidine chase to become fully effective, and the effectiveness of the chase may have differed between species or cell types. For example, increasing the concentration of the thymidine chase from 25 μM to 100 μM significantly decreased the number of nuclei in the ‘arm’ for maize root tip samples (Supplementary Fig. S1), but residual incorporation occurred even with a chase concentration as high as 200 μM in wheat root tips (Supplementary Fig. S2C). It is important to note that EdU labeling was displayed on a logarithmic scale in the flow cytograms and most of the residual labeling was 10- to 100-fold lower than that of fully labeled nuclei. Furthermore, the residually labeled nuclei were excluded from the gates used to follow the DNA content of pulse-labeled nuclei, and did not affect the RM measurements described below or the resulting estimates of S-phase duration.

### Relative movement and measurement of S-phase duration

To quantify progression through S phase, we drew gates around the three populations of G_1_, G_2_/M, and S-phase (EdU-labeled) nuclei and calculated the mean DAPI fluorescence, a measure of DNA content, for each population. The RM of the nuclei in S phase was then calculated for each time point (refer to the Materials and methods for gating and RM calculations). Assuming that the increase in DNA content occurred at a constant rate throughout S phase, the average DNA content of labeled nuclei at 0h should be half way between 2C and 4C, with an RM of 0.5 (Begg *et al.*, 1985). In theory, the RM should continue to increase as nuclei progress through S phase, with a value of 1.0 being reached when all nuclei that initiated DNA synthesis during the labeling period finished replication (Begg *et al.*, 1985).

**Fig. 2.**
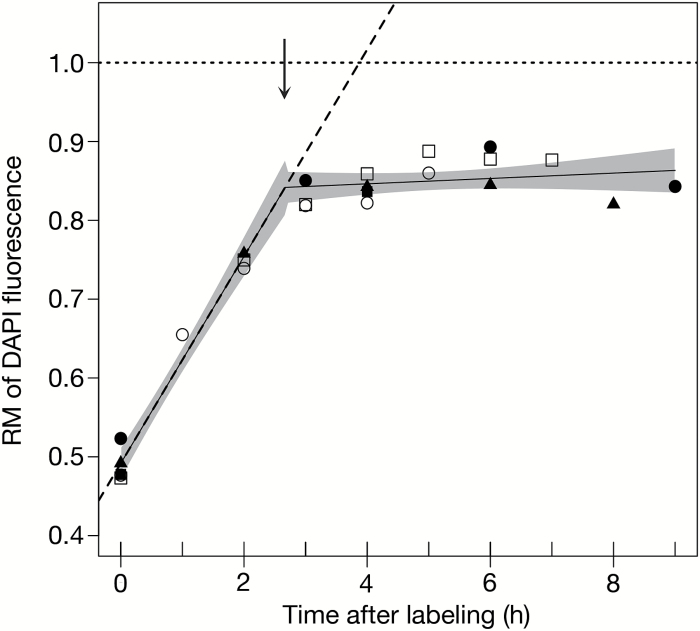
Relative movement plot and estimating S-phase duration in maize root tip cells. The relative movement (RM) of labeled nuclei at each time point was calculated using the equation RM=(F_L_–F_G1_)/(F_G2/M_–F_G1_) (Begg *et al.*, 1985). The RM values from multiple biological replicates were combined and plotted as a function of time after EdU labeling. Biological replicates are indicated on the RM plot as follows: biological replicate 1, filled circle; 2, open circle; 3, filled square; 4, open square; and 5, filled triangle. A segmented linear regression analysis was used to fit two component lines (black lines) with a single breakpoint to the data of combined biological replicates. The 95% confidence intervals for the slopes of the two fitted lines are shaded in gray and the *R* ^2^ value for the segmented fit is 0.98. A lower S-phase duration estimate, 2.7h, was determined by the *x*-axis value at the breakpoint (vertical, downward arrow) and represents the average duration for the largest cohort of proliferating nuclei in maize root tips. The slope of the first linear component (*y*=0.1312*x*+0.4917) made up of data points below the breakpoint represents the initial rate of DNA replication. Extrapolation of this line (dashed line) to an RM of 1.0 (dotted line) gave a second, upper estimate of 3.9h for S-phase duration and accounts for more slowly proliferating nuclei.

**Fig. 3.**
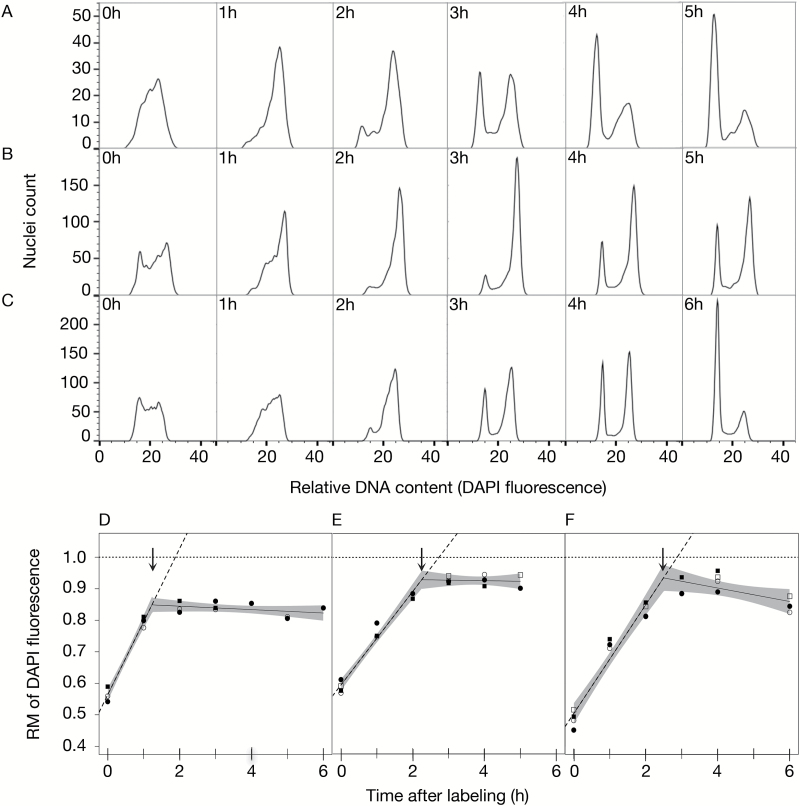
Estimation of S-phase duration in rice, barley, and wheat root tip cells. Roots from 4-day-old rice seedlings, 2-day-old barley seedlings, and 3-day-old wheat seedlings were pulse-labeled with 25 μM EdU for 30min, washed, and then chased with varying concentrations of thymidine (refer to the Materials and methods for details) for select hourly intervals. Terminal 5mm root segments were excised, fixed, and frozen. Nuclei were prepared and analyzed by flow cytometry as described in Fig. 1. (A–C) Histograms of relative DNA content of S-phase nuclei from an S-phase duration time course for (A) rice, (B) barley, and (C) wheat. (Corresponding flow cytograms are in Supplementary Fig. S2.) Data represent single biological replicates. (D–F) RM plots for (D) rice, (E) barley, and (F) wheat root tip cells. For each species, RM values from multiple biological replicates were calculated and plotted as a function of time. Segmented regression analysis was applied to the data of combined biological replicates as described in Fig. 2. The *R* ^2^ value of the segmented fit, slope of the first component line, and lower and upper average S-phase duration estimates are as follows: (D) rice, *R* ^2^=0.98, *y*=0.2318*x*+0.5635 and 1.2h/1.9h; (E) barley, *R* ^2^=0.98, *y*=0.1488*x*+0.5937 and 2.3h/2.7h; and (F) wheat, *R* ^2^=0.95, *y*=0.1727*x*+0.5038 and 2.5h/2.9h.

**Fig. 4.**
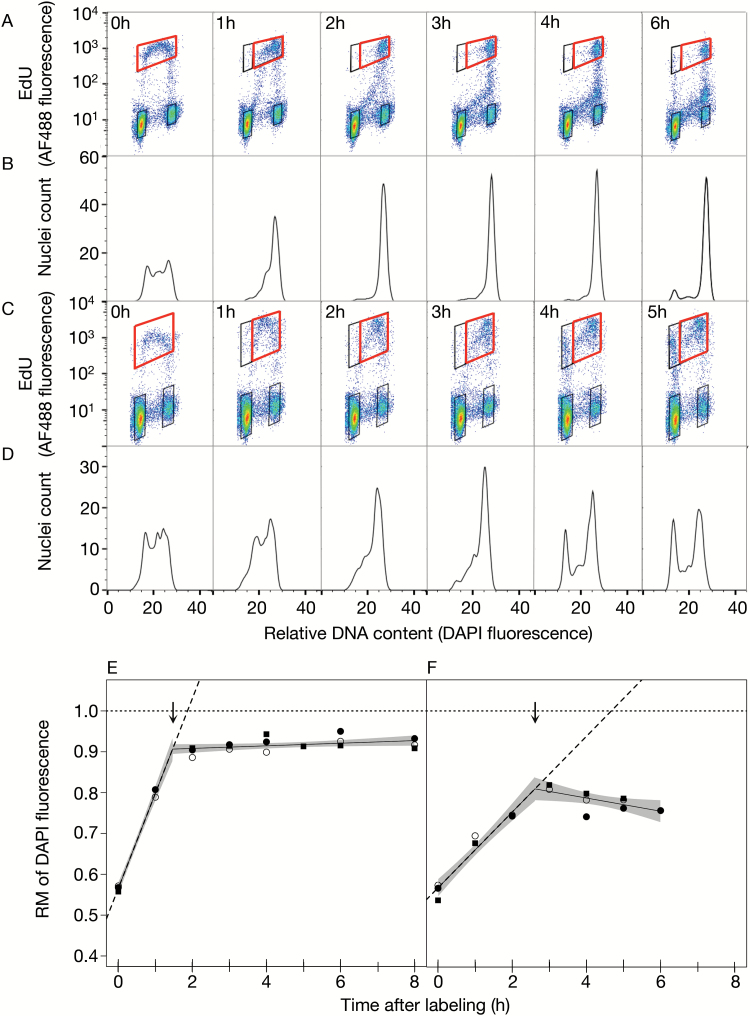
Flow cytometric analysis and estimation of S-phase duration in Arabidopsis and rice suspension cell cultures. Four (Arabidopsis) or three (rice) days after transfer, cells in logarithmic growth phase were pulse-labeled with 10 μM (Arabidopsis) or 25 μM (rice) EdU for 30min and then chased with 100 μM thymidine for select hourly intervals. Cells were pelleted, fixed, washed, and frozen. Nuclei were prepared and analyzed by flow cytometry as described in Fig. 1. (A–D) Bivariate flow cytograms and corresponding histograms of relative DNA content of S-phase nuclei isolated from cell suspension cultures of (A, B) Arabidopsis and (C, D) rice. Data represent single biological replicates. (E, F) RM plots for (E) Arabidopsis and (F) rice cell cultures. For each species, RM values from multiple biological replicates were calculated and plotted as a function of time. Segmented regression analysis was applied to the data of combined biological replicates as described in Fig. 2. The *R* ^2^ value of the segmented fit, slope of the first component line, and lower and upper average S-phase duration estimates are as follows: (E) Arabidopsis, *R* ^2^=0.99, *y*=0.2326*x*+0.5654 and 1.5h/1.9h; and (F) rice, *R* ^2^=0.95, *y*=0.0926*x*+0.5676 and 2.6h/4.7h.

In our experiments, RM values at 0h (samples taken immediately after the 30min labeling period) ranged from 0.45 to 0.61, essentially as predicted (Figs 2, 3D–F, 4E, F; 0h). RM values also increased over time as expected, although, in the actively growing systems we studied, they did not reach the theoretical value of 1.0. This value would only have been reached if the labeled cells all accumulated in G_2_ without returning to G_1_ during the time course of the experiment, as apparently was the case in the experiments of Begg and colleagues with BrdU-labeled CHO cells (Begg *et al.*, 1985). In our calculations, we minimized the effect of G_1_ return by excluding EdU-labeled nuclei with 2C DNA content (Fig. 1A; 2–7h, gray boxes). However, labeled nuclei that failed to complete S phase or that returned to G_1_ and subsequently began a second round of DNA synthesis caused the RM values to plateau at an RM value <1.0.

To estimate the point at which a plateau was reached, we applied a segmented linear regression analysis in which two linear components and a single breakpoint were fit to the data. To ensure high confidence fitting of the linear regression, data from multiple biological replicates were combined prior to statistical analysis. The precision of the analysis was indicated by 95% confidence intervals on the slopes of each of the component lines (Figs 2, 3D–F, 4E, F). Two estimates of S-phase duration were obtained from this analysis. One estimate involved taking the slope of the first linear component as the initial rate of DNA replication and extrapolating the line to an RM value of 1.0. A second estimation procedure assumed that the breakpoint in the RM curve represents essentially complete replication of the predominant cell population. The breakpoint estimate probably best reflects the behavior of the majority of cells with labeled nuclei because it minimizes the effect of cells that failed to progress through S phase, as well as the effect of cells that returned to G_1_ and began a second round of synthesis. We cannot formally distinguish between these two possibilities, which are not mutually exclusive. However, a second round of DNA synthesis in a subset of the labeled cells seems quite likely in a rapidly dividing population such as the root tips used here. Initiating a second round of DNA synthesis within the time frame of our experiments would require G_1_ to be traversed quite rapidly, but brevity of G_1_, or even its absence, is well documented in rapidly dividing animal cells (White and Dalton, 2005; Orford and Scadden, 2008), and G_1_ durations between 0.5h and 1.0h have been documented in root meristems of monocots (Evans and Rees, 1971).

The following paragraphs describe our analyses for several different species. S-phase duration estimates and RM values at the breakpoint for each species studied are summarized in Table 1.

**Table 1.** Average S-phase duration estimates with corresponding breakpoint RM values, genome size, and tissue source

**Species**	**Tissue type**	**S-phase duration breakpoint/extrapolated (h**)	**RM value at breakpoint**	**Genome size (Gbp**)
**Maize cv. B73**	Roots	2.7/3.9	0.85	2.3 (Schnable *et al.*, 2009)
**Barley cv. Morex**	Roots	2.3/2.7	0.94	5.1 (Mayer *et al.*, 2012)
**Wheat cv. Chinese Spring**	Roots	2.5/2.9	0.94	17 (Brenchley *et al.*, 2012)
**Rice cv. Nipponbare**	Roots	1.2/1.9	0.84	0.39 (Matsumoto *et al.*, 2005)
**Rice cv. Nipponbare**	Culture	2.6/4.7	0.81	0.39 (Matsumoto *et al.*, 2005)^*a*^
**Arabidopsis (Col 0**)	Culture	1.5/1.9	0.91	0.13 (Arabidopsis Genome Initiative, 2000)^*a*^

^*a*^ Genome sizes are known to vary in tissue culture (Lee and Phillips, 1988).

### S-phase duration in root tips

Immediately after EdU labeling, S-phase nuclei from maize root tips were distributed evenly between 2C and 4C (Fig. 1A, B; 0h). Through 4h, there was a steady increase in the amount of labeled nuclei that reached 4C. At some point between 3h and 4h, labeled nuclei began to return to 2C. At subsequent time points, the number of labeled nuclei at 2C steadily increased and those at 4C slowly decreased. As discussed above, it is likely that some of these returned, labeled nuclei initiated a second round of DNA synthesis, resulting in a steady-state population of labeled nuclei with intermediate DNA content and lowered the RM value at the breakpoint (0.85) (Fig. 2; Table 1). The effect was to increase the difference between the breakpoint (2.7h) and extrapolated (3.9h) estimates of S-phase duration (Fig. 2). These observations suggested that a large fraction of maize root tip nuclei completed S phase in ~2.7h.

Histograms from time course analyses with root tips of other grass species are presented in Fig. 3 (see Supplementary Fig. S2 for flow cytograms). In rice (Fig. 3A), a large fraction of labeled nuclei approached 4C after a 1h chase period. Between 1h and 2h, a small fraction of labeled nuclei returned to 2C and the fraction of labeled nuclei in 2C significantly increased between 2h and 3h. Like maize, the rice RM value at the breakpoint, 0.84 (Fig 3D; Table 1), was relatively low, most probably because of labeled nuclei undergoing a second round of replication. As a consequence, the two estimates of S-phase duration (1.2h and 1.9h) were different. We suggest that the largest cohort of rice root tip nuclei completed replication in 1.2h (Fig. 3D).

In barley (Fig. 3B), a large fraction of the labeled nuclei reached 4C by 3h and return of labeled nuclei to 2C was apparent between 2h and 3h. A significant fraction of labeled nuclei had returned to 2C by 4h. Some of these may have undergone a second round of replication, but the effect was smaller than in maize or rice. Thus, the RM value at the breakpoint, 0.94 (Fig. 3E; Table 1), was closer to 1.0 and the breakpoint and extrapolation estimates of S-phase duration, 2.3h and 2.7h, respectively, were more similar (Fig. 3E).

In wheat (Fig. 3C), a large population of labeled nuclei reached 4C at ~2–3h and a substantial fraction returned to 2C by 3h. As in barley, the RM value at the breakpoint, 0.94 (Fig. 3F; Table 1), was high and the two estimates of S-phase duration, 2.5h and 2.9h, were similar (Fig. 3F).

### S-phase duration in suspension cultures

In Arabidopsis cultured cells (Fig. 4A, B), a large fraction of labeled nuclei reached 4C between 1h and 2h. However, the return of labeled nuclei to 2C occurred between 4h and 6h, in contrast to 2–4h in the root tip samples, and probably accounted for the decreased numbers of labeled nuclei with intermediate DNA contents in the flow cytograms (Fig. 4A). This observation is not too surprising given that cultured plant cells often have an extended cell cycle duration (refer to table 1 in Gould and King, 1984; and table 8 in Grif *et al.*, 2002) with G_1_ duration considerably longer than that of root tips (Gould and King, 1984). Returning nuclei, therefore, had a smaller influence on RM calculations, although there may have been a small number of nuclei progressing slowly or perhaps even arrested in mid-S phase. The RM value for the Arabidopsis cells at the breakpoint, 0.91 (Fig. 4E; Table 1), was high and both 1.5h and 1.9h are good estimates of S phase for the largest cohort of proliferating nuclei (Fig. 4E).

In rice cell culture, a large cohort of labeled nuclei reached 4C by 3h (Fig. 4C, D) and a significant return to 2C occurred between 3h and 4h. Notably, a large subpopulation of nuclei either appeared to arrest part way through S phase or proceeded much more slowly than the main cohort of labeled nuclei. This subpopulation is clearly visible in the flow cytograms at 3–5h and is visualized as a pronounced shoulder on the 4C peak in the corresponding histograms (Fig. 4C, D). The subpopulation lowered the RM value at the breakpoint, 0.81 (Fig. 4F; Table 1), and resulted in a large difference between the two S-phase estimates, 2.6h and 4.7h (Fig. 4F). In this case, 4.7h is probably a considerable overestimate of S-phase duration for the major class of labeled nuclei, and 2.6h more accurately reflects the S-phase duration for this group.

## Discussion

We sought a technique for directly estimating S-phase duration in large populations of cells without the need for disruptive synchronization treatments. Thus, we used bivariate flow cytometry to follow the increase in DNA mass that occurs between G_1_ and G_2_ using EdU incorporation as a tool to focus on a cohort of cells actively engaged in DNA synthesis. Our method can be applied to any system containing a sufficient number of cells that can be labeled with EdU and can be analyzed by flow cytometry as either cells or nuclei. In our hands, the method is highly reproducible and reduces the complexity of obtaining robust estimates of S-phase duration for large populations of cells.

The root tip system and EdU labeling techniques are applicable to a wide variety of plant species. Root tips efficiently take up both EdU and thymidine, and contain a large fraction of proliferating cells. Advantages of EdU labeling include the fact that visualization of incorporated EdU is achieved rapidly and under mild conditions that do not degrade nuclei or subnuclear structure (Kotogany *et al.*, 2010). Our flow cytometric procedure provides a direct measurement of the increase in DNA mass that occurs during replication and can easily be applied to unsynchronized cell populations. As such, it eliminates the necessity for disruptive synchronization treatments such as sucrose starvation or addition of chemical inhibitors (Dolezel *et al.*, 1999; Planchais *et al.*, 2000; Menges and Murray, 2002) and can be applied to nuclei derived from cell cultures as well as intact organs. Large numbers of nuclei can be analyzed rapidly, offering increased statistical power compared with techniques that require scoring of individual nuclei under the microscope. Microscopic techniques will continue to be useful in many instances, especially in cases where material is limited and/or where it is important to analyze S phase in an anatomically defined subset of cells. However, flow cytometry is advantageous in systems in which large numbers of cells or nuclei from proliferating cell populations can be isolated for analysis.

Bivariate flow cytometric analysis of the cell cycle using BrdU has been used to follow DNA synthesis in both plant (Lucretti *et al.*, 1999) and animal (Begg *et al.*, 1985) systems. Begg and colleagues applied this technique to estimate the duration of S phase, using RM plots similar to ours. Under their conditions with mammalian cells, RM values approached 1.0 as the population of labeled cells completed a single round of replication. However, in our material, some labeled nuclei initiated a second round of DNA synthesis and, in some cases, a subset of nuclei progressed slowly or arrested prior to completion of S phase. The presence of one or both of these two types of intermediate DNA contents lowered RM values and prevented them from reaching 1.0 as was achieved in the mammalian systems. In all cases in this study, the RM plots showed a clear break in slope before reaching 1.0, and this led us to apply a segmented linear regression analysis to fit two components to our data and to determine the breakpoint between them. We interpreted the breakpoint as representing the time at which the largest cohort of nuclei completed DNA synthesis. In most cases, we think this is the best estimate of S-phase duration for the population under study, a view supported by detailed analysis of flow cytometry profiles presented in the Results.

### Cells versus roots

Many features of the cell culture environment are known to affect total cell cycle length and it is well documented that suspension cell cultures typically have a much longer G_1_ phase than root meristems (Gould and King, 1984, and references therein). Less is known about the effect of culture parameters on the S-phase component of the cell cycle. The fact that S-phase length estimates vary as much as 2- to 3-fold between different studies using suspension cells from the same species suggests that S-phase progression is likely to be sensitive to multiple environmental influences (see references for *Acer pseudoplatanus* and *Haplopappus gracilis* in table 1 of Gould and King, 1984).

We compared S-phase duration for cultured cells and root meristems in both rice and Arabidopsis. Unfortunately, we could not obtain data of sufficient quality from Arabidopsis root tips (see Supplementary Fig. S3 at *JXB* online). However, comparison of rice suspension culture cells with rice root tip meristem cells showed that S phase is shorter in root tips. RM plots for suspension cells showed a lower slope of the first component line (compare Figs 4F and 3D), indicative of the presence of a slower replicating group of cells in addition to the main cohort. Extrapolating from the first component to estimate an overall average S phase for suspension cells and root tips gave values of 4.7h and 1.9h (Table 1), respectively, for a 2.5-fold difference. Estimates derived from breakpoint values, which reflect the most rapidly replicating major cohort of nuclei, differed by more than a factor of two (2.6h for suspension cells and 1.2h for root tips; Table 1).

The presence of a more slowly proliferating subset of cells in plant cell suspension cultures may reflect that many such cultures are comprised of clumps or multicellular aggregates alongside isolated, single cells. Cells in the interior of aggregates are subject to a different microenvironment compared with free floating cells or cells on the surface of aggregates (Street, 1973), including differences in oxygen (Hulst *et al.*, 1989) and nutrient availability (Patil and Roberts, 2013). Other factors that may contribute to the difference in S-phase duration include genetic and epigenetic instability, such as chromosome rearrangements, DNA methylation, and somatic mutation (reviewed by Lee and Phillips, 1988; Phillips *et al.*, 1994; Tanurdzic *et al.*, 2008).

### Comparison with other reported estimates

We compared our S-phase duration estimates with previously reported estimates for species included in our study (Supplementary Table S1). There is a clear tendency for our estimates to be shorter than those in previous reports. There are several explanations for the differences. Because flow cytograms do not cleanly resolve nuclei that have replicated 100% of their DNA from those having replicated slightly less than 100%, the breakpoint method may underestimate the full duration of S phase, especially in cases where a subset of the DNA (e.g. maize knob heterochromatin or B chromosomes) replicates extremely late in S phase. The extrapolation method is less sensitive to this problem but can significantly overestimate duration when a fraction of cells fail to complete S phase, as observed in our rice suspension culture. In other cases, the breakpoint and extrapolation methods may be thought of as yielding lower and upper estimates of S-phase duration, respectively.

Other reasons for variation in S-phase estimates include the fact that different subsets of cells within the root meristem can vary in S-phase duration (Barlow and Macdonald, 1973; Van’t Hof, 1974, and references therein; Hayashi *et al.*, 2013). Some techniques focus on certain groups of cells, while our method provides an estimate of the average duration for the major cohort of cells in S phase. Temperature and a variety of other environmental variables are also known to affect S-phase duration (Van’t Hof, 1974, and references therein; Verma, 1980; Francis and Barlow, 1988; Grif *et al.*, 2002). Older estimates may have been influenced by technical factors inherent to the methodology, such as radiological effects (De La Torre and Clowes, 1974; reviewed in Gould and King, 1984; Darzynkiewicz *et al.*, 1987; Fiorani and Beemster, 2006) as well as sampling errors and/or mathematical assumptions (Shackney and Ritch, 1987; Grif *et al.*, 2002). Considering the many factors that can affect the duration of S phase, it is evident that measurement of S should be made under conditions that closely match those of other experiments with which they will be compared. Our method was designed to match the experimental conditions we use to study replication timing programs but can accommodate a wide range of other conditions.

S-phase duration estimates in other eukaryotes exhibit similar within-species variability as has been observed for plants (Supplementary Table S2). More diverse cell types have been studied in mammalian systems such as mouse, rat, and human, whereas in plants most reports focus on roots. The broader sampling of tissue types and cell lines in mammals may explain the discovery of a few examples of notably long S-phase estimates in some specialized tissues or lines.

### Relationship to genome size

We estimated the average S-phase duration in root tips from a selection of grass species with widely varying genome sizes to address the question as to whether S-phase duration was correlated to genome size (Table 1). The selected species cover a 40-fold range in genome size. However, comparison of the breakpoint estimates of S-phase duration only uncovered an ~2-fold difference in the duration of S phase. When both the breakpoint and extrapolation estimates of S-phase duration are considered, there is an ~3-fold difference in the duration of S phase. The lack of an obvious relationship of S-phase duration and genome size was initially surprising given early reports that used the PLM method to show a trend toward longer S phases in species with larger genome sizes (Van’t Hof, 1965; Evans and Rees, 1971). The significance of this trend depended heavily on the inclusion of species from such genera such as *Allium* and *Tradescantia* with very large genomes in the range of 20–33 Gbp. In contrast, plants with moderately sized genomes showed only small, apparently random, variations in S-phase duration. Similarly, the sampling of previously reported plant S-phase duration values in Supplementary Table S1 shows no correlation to genome size. Several studies (reviewed by Bennett, 1972; Francis *et al.*, 2008) reported a strong dependence of total cell cycle time on genome size. However, given that DNA replication occurs in separate replicons, many of which can replicate simultaneously, S-phase duration may not be strongly dependent on genome size.

## Supplementary data

Supplementary data are available at *JXB* online

**Figure S1.** Flow cytometric analysis of maize roots chased with 100 μM thymidine.

**Figure S2.** Flow cytometric analysis of rice, barley, and wheat root tips.

**Figure S3.** Hydroponic growth system, flow cytometric analysis, and DNA content histograms of S-phase nuclei from roots of 4-day-old Arabidopsis seedlings.

**Table S1.** Comparison of S-phase duration estimates in plants.

**Table S2.** S-phase duration estimates in various eukaryotes.

Supplementary Data

## Abbreviations:

BrdU: 5-bromo-2'-deoxyuridine
CLB: cell lysis buffer
EdU: 5-ethynl-2'-deoxyuridine
FSC: forward scatter
PBS: phosphate-buffered saline
RM: relative movement
SSC: side scatter.
