# Supplementary material for: A flow cytometric method for estimating S-phase duration in plants
Source: J Exp Bot. 2016 Oct 3;67(21):6077–87. doi: 10.1093/jxb/erw367 (PMC5100020; doi:10.1093/jxb/erw367)
Supplement: Supplementary Data [file supp_67_21_6077__index.html]

A flow cytometric method for estimating S-phase duration in plants — A flow cytometric method for estimating S-phase duration in plants — Supplementary Data 

# A flow cytometric method for estimating S-phase duration in plants

## Supplementary Data

Data files

- supplementary\_figures\_S1\_S3.pdf - Supplementary Data
- supplementary\_tables\_S1\_S2.pdf - Supplementary Data
