# Supplementary material for: A flow cytometric method for estimating S-phase duration in plants
Source: J Exp Bot. 2016 Oct 3;67(21):6077–87. doi: 10.1093/jxb/erw367 (PMC5100020; doi:10.1093/jxb/erw367)
Supplement: Supplementary Data [file supp_erw367_supplementary_figures_S1_S3.pdf]

Supplementary Figure S1.

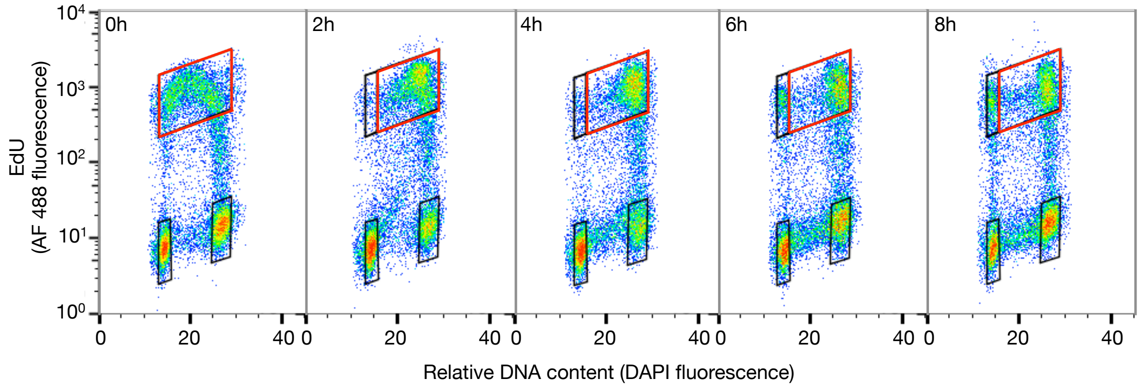

Figure S1. Flow cytometric analysis of maize roots chased with 100 µM thymidine. Roots of three-day old maize seedlings were pulse-labeled with 25 µM EdU for 30 min, washed, and then chased with 100 µM thymidine for select hourly intervals. Terminal 1 mm segments of the root tips were excised, fixed and frozen. Nuclei were prepared and analyzed by flow cytometry as described in Fig. 1. Data represent a single biological replicate. The "arm" of nuclei present in flow cytometry plots of maize roots chased with 25 µM thymidine (Fig. 1A) was greatly diminished when chasing with 100 µM thymidine. Note that these lower level labeled nuclei in the "arm" were excluded from gates used to follow the DNA content of pulse-labeled nuclei and did not affect RM measurements or estimates of S-phase duration.

Supplementary Figure S2.

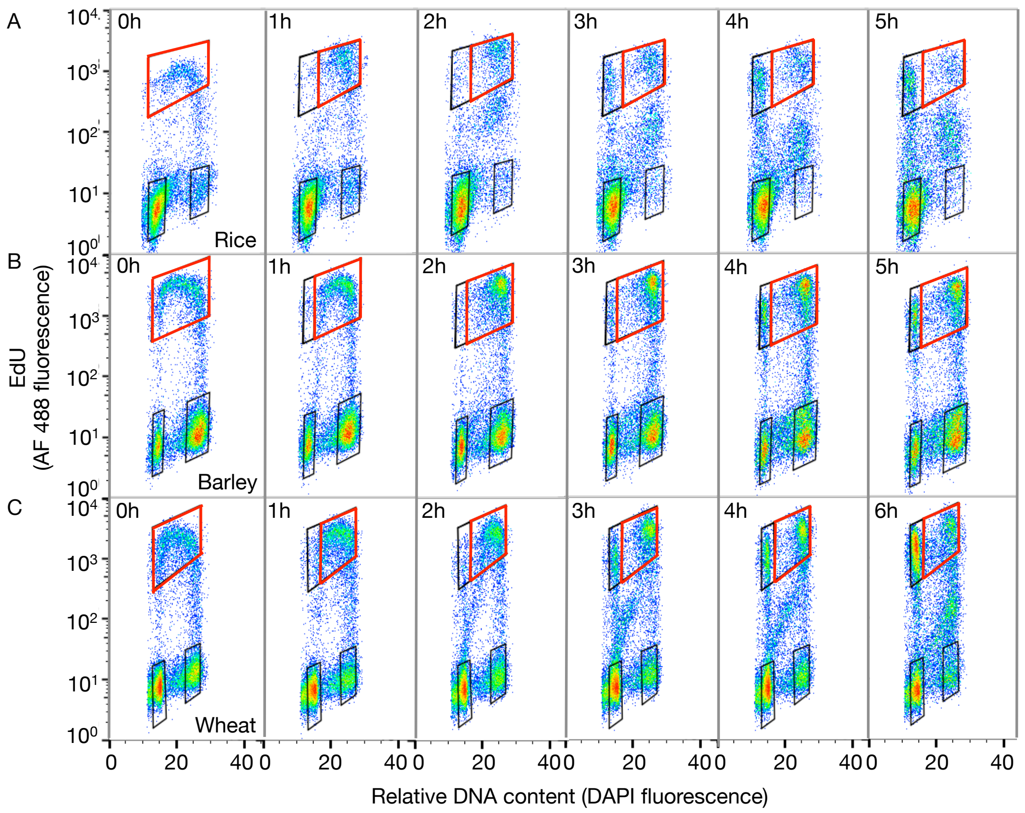

Figure S2. Flow cytometric analysis of rice, barley and wheat root tips. Roots from four-day old rice seedlings, two-day old barley seedlings, and three-day old wheat seedlings were pulse-labeled with 25 μM EdU for 30 min, washed, and then chased with varying concentrations of thymidine (refer to Materials and methods for details) for select hourly intervals. Terminal 5 mm segments of the root tips were excised, fixed and frozen. Nuclei were prepared and analyzed by flow cytometry as described in Fig. 1. Data represent a single biological replicate. (A-C) Bivariate flow cytograms for (A) rice, (B) barley, and (C) wheat root tips.

Supplementary Figure S3.

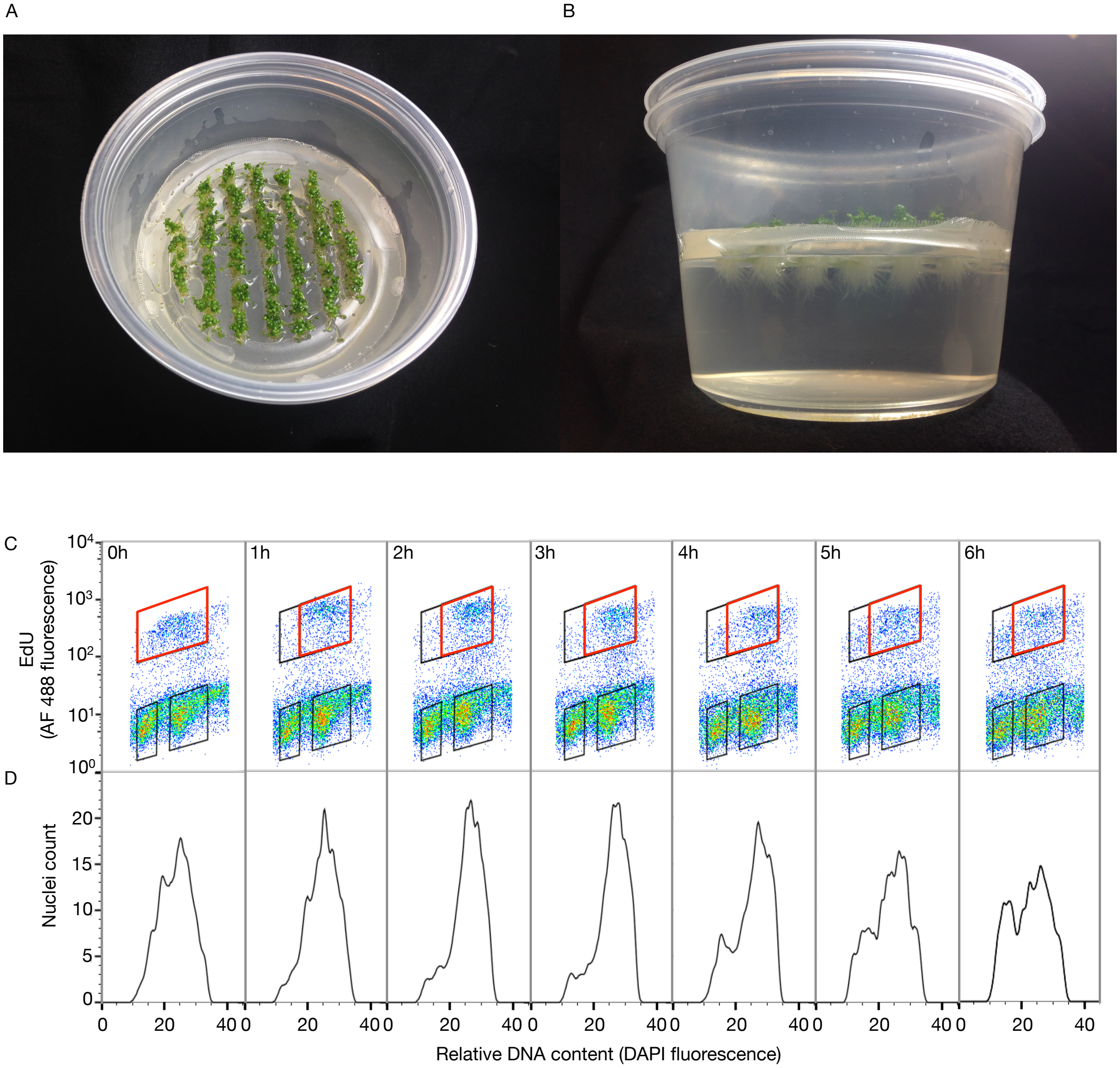

Figure S3. Hydroponic growth system, flow cytometric analysis and DNA content histograms of S-phase nuclei from roots of 4-day old Arabidopsis seedlings. (A) Arabidopsis seeds were germinated using a hydroponic system described by Alatorre-Cobos *et al*. (2014). (B) An example of the size of the 4-day old roots used for EdU labeling is depicted. Four-day old seedling roots were pulse-labeled with 10 µM EdU for 30 min, washed, and then chased with 100 µM thymidine for select hourly intervals. Roots were fixed and terminal 3-5 mm root segments were excised and frozen (refer to Materials and methods for details). Nuclei were prepared and analyzed by flow cytometry as described in Fig. 1. (C) Bivariate flow cytograms and (D) corresponding DNA content histograms of S-phase nuclei. Data represent a single biological replicate. We were unable to use these data to make reliable estimates of S-phase

duration due to the low quality of the nuclear preparations, which resulted in broadened peaks and overwhelming amounts of cellular debris. Much of this debris could not effectively be excluded using SSC vs. 460/50 nm plots because their fluorescence fell within the same range as the cycling nuclei. As a result, it was difficult to accurately gate the labeled nuclei in bivariate 530/40 nm vs. 460/50 nm plots. One or a combination of technical optimizations may help to improve the flow cytometry data quality for Arabidopsis roots, such as the age of seedlings, length of excised roots, grinding conditions, or application of a Percoll gradient purification step. We found the extremely fine roots of young Arabidopsis seedlings were not as amenable to the bulk nuclei isolation procedure from fixed tissue used in this study and it may be that a different nuclei isolation approach is needed for plants with similarly fine roots.
