# Supplementary material for: A flow cytometric method for estimating S-phase duration in plants
Source: J Exp Bot. 2016 Oct 3;67(21):6077–87. doi: 10.1093/jxb/erw367 (PMC5100020; doi:10.1093/jxb/erw367)
Supplement: Supplementary Data [file supp_erw367_supplementary_tables_S1_S2.pdf]

Supplementary Table S1. Comparison of S-phase duration estimates in plants. This is a comparison of the reported estimates of S-phase duration of plants used in this study and illustrates the tendency of our estimates of S-phase duration to be shorter than most other reported estimates. It shows the variation of S-phase duration estimates reported within a given species and also shows the relative conformity of the reported S-phase duration values among all species, especially given the range in genome sizes.

Species	Genome Size (Gbp)[a]	S-phase Duration (h)	Tissue	Temperature (°C)	Method for Determining S-phase Duration	Citation[b]	Notes
MAIZE	2.3						
Zea mays cv. B73		2.7/3.9	Roots	28	RM/Flow Cytometry	This paper	Breakpoint/Extrapolation
Zea mays cv. Seneca		2.5-8	Roots	20-35	PLM	Verma, 1980	Different temperatures (20, 25, 30, 35 °C)
Zea mays cv. Seneca		4.8	Roots	20	PLM	Kidd *et al.*, 1987	
Zea mays		4.5-5.5	Roots	25	PLM	Verma and Lin, 1979	Different genetic stocks (Seneca 60, 9-tester, W23, KYS) and different W23 "auto" ploidies
Zea mays (B73xMo17)		~5	Roots	RT	HU/Flow cytometry	Lee *et al.*, 1996	HU removed by washing
Zea mays		4.25	Roots	-	PLM	Evans and Rees, 1971	
Zea mays cv. Golden Bantam		2.7-16	Roots	21	PLM	Barlow and Macdonald, 1973	Study of different tissue types and developmental variation
Zea mays cv. Golden Bantam		10.2	Roots	20-22	PLM	Olszewska *et al.*, 1990	
Zea mays		11.5	Roots	22	PLM	Gahan and Hurst, 1976	Study of age variation in seedling roots, 1-20 days
Zea mays cv. Black Mexican Sweet		7.0-14	Roots, culture	27	PLM	Wang *et al.*, 1986	Effect of B chromosomes, cell culture
Zea mays		5.0-14.8	Roots	22-25	PLM	Grif *et al.*, 2002 and references therein	Different ploidies and nuclear DNA amounts; Review, multiple citations
RICE	0.39						
Oryza sativa cv. Nipponbare		1.2/1.9	Roots	28	RM/Flow Cytometry	This paper	Breakpoint/Extrapolation
Oryza sativa cv. IR 34		3	Roots	30/20	PLM	Kidd *et al.*, 1987	Grown at 30C and equilibrated to 20C prior to labeling
Oryza sativa		5.6-6	Roots	27	PLM	Grif *et al.*, 2002 and references therein	K-3968, K-4996; Review, multiple citations
Oryza sativa cv. Nipponbare		2.6/4.7	Cells 3-d after transfer	27	RM/Flow Cytometry	This paper	Breakpoint/Extrapolation
BARLEY	5.1						
Hordeum vulgare cv. Morex		2.3/2.7	Roots	28	RM/Flow Cytometry	This paper	Breakpoint/Extrapolation
Hordeum vulgare var. *hakata*		6	Roots	25	PLM	Kusanagi, 1966	

Species	Genome Size (Gbp)[a]	S-phase Duration (h)	Tissue	Temperature (°C)	Method for Determining S-phase Duration	Citation[b]	Notes
Hordeum vulgare var. *sultan*		6	Roots	20	PLM	Kidd *et al.*, 1987	
Hordeum vulgare		3.5,3.8	Roots	20	PLP	Bennett and Finch, 1972	Varieties *maris otter* and *sultan*
WHEAT	17						
Triticum aestivum cv. Chinese Spring		2.5/2.9	Roots	23	RM/Flow Cytometry	This paper	Breakpoint/Extrapolation
Triticum aestivum var *maris ranger*		8.6	Roots	20	PLM	Davies and Rees, 1975	
Triticum aestivum		4.8	Roots	-	PLM	Francis *et al.*, 2008	
Triticum aestivum		3.2-7	Roots/ Shoots	22-25	PLM	Grif *et al.*, 2002 and references therein	Review, multiple citations
Triticum aestivum var *indus*		8	Roots	23	PLM	Evans and Van't Hof, 1975	
ARABIDOPSIS	0.13						
Arabidopsis thaliana (Nossen)		2.9, 8.7	Roots	22	*In Situ* Labeling	Hayashi *et al.*, 2013	Meristem, elongation zone
Arabidopsis thaliana		2	Roots	22	*In Situ* Labeling	Hu *et al.*, 2015	
Arabidopsis thaliana (Col 0)		2.8	Seedlings	22	PLM	Van't Hof *et al.*, 1978	
Arabidopsis thaliana		2.9	Leaves	22	Kinematics/Flow cytometry	Dhondt *et al.*, 2010	
Arabidopsis thaliana (Col 0)		1.5/1.9	Cells 4-d after transfer	23	RM/Flow Cytometry	This paper	Breakpoint/Extrapolation
Arabidopsis thaliana (Col-0)		2.5-12	Cells	-	Cell doubling time/flow cytometry	Richard *et al.*, 2001	1-5 days after cell transfer
Arabidopsis thaliana (Landsberg) MM2		3	Cells 3-d after transfer	27	Cell doubling time/flow cytometry	Menges *et al.*, 2006	

[a]Genome size citations in main text Table 1. [b]Citation references listed below. HU=hydroxyurea; PLM=percent labeled mitosis; PLP=percent labeled prophases; RM=relative movement

Supplementary Table S2. S-phase duration estimates in various eukaryotes. This table is a non-exhaustive sampling of the reported estimates of S-phase duration in diverse eukaryotic cell and tissue types and illustrates the large amount of variation within and between species, and occasionally disagreement even amongst the reported values for a single cell type. Approximate genome sizes for each species are presented for reference.

Species	Genome Size (Gbp)[a]	Citation for Genome Size[b]	S-phase Duration	Cell/Tissue	Method for Determining S-phase Duration	Citation[b]
Caenorhabditis elegans (nematode)	0.1	Hillier *et al*., 2005	1.3-2.9 h	Gonads (mitotic and meiotic S)	Double labeling assay	Jaramillo-Lambert *et al.,* 2007
Drosophila melanogaster (fruit fly)	0.1	Adams *et al*., 2000	3-4 min	Embryo (cycle 1-13)	Electron microscopy/Rate of replication	Blumenthal e*t al*., 1974
			3.5 h	Ganglial neuroblast cells, 3rd instar larvae	3H-thymidine labeling and autoradiography	Trunova *et al*., 1998
			3.4-50 min	Embryo (cycle 2-14)	GFP-PCNA labeling/FM	Shermoen *et al*., 2010
			3 h	Wing imaginal disc	BrdU/FCM	Dubatolova *et al*., 2011
Gallus gallus (red junglefowl/chicken)	1.0	Hiller *et al*., 2004	5-6 h	Various epithelium tissues	PLM	Cameron, 1964
Rattus norvegicus (brown rat)	2.8	Gibbs *et al*., 2004	2-4 h	Various embryonic tissues	Nocodazole/FCM	Macauley *et al*., 1993
			9 h	Olfactory epithelium globose basal cells	Double labeling assay	Huard and Schwob, 1995
			4.6 h	Neural progenitor cells	Serial BrdU labeling/FM	Zhang *et al*., 2006
Mus musculus (mouse)	2.5	Waterston *et al*., 2002	30 h	Ear epidermis	PLM	Sherman *et al.,* 1961
			6.9-7.5 h	Various epithelium tissues	PLM	Cameron and Greulich, 1963
			6.5-7.1 h	Yolk sac and placenta tissues	PLM	Cameron, 1964
			13.5 h	Forestomach epithelium	PLM	Wolfsberg, 1964
			7 h	Oocyte, 1st cell cycle	Synchronization/microdensitometry	Howlett and Bolton, 1985
			7.6 h	Intestine jejunal crypts	Double labeling assay	Abe *et al*., 1992
			14 h	Mammary epithelium	Double labeling assay	Christov *et al*., 1993
			4.8 h	Whole cultured embryos	Serial BrdU labeling/FM	Gressens *et al*., 1998
			7.1 h	Tibial growth plate	Double labeling assay	Vanky *et al*., 1998
			6.3-8.3 h	Cerebral wall (in culture)	Double labeling assay	Takahashi *et al*., 1999
			3-5 h	Epidermis	Double labeling assay	Barthel *et al*., 2000
			12.9 h	Neural stem & progenitor cells	Double labeling assay	Farioli-Vecchioli *et al*., 2014
			4.9 h	3T3 fibroblasts cell line	HU synchronization/FCM	Sladek and Jacobberger, 1992
			7-8 h	Bone marrow cell line	PLM	Reddy *et al*., 1997

Species	Genome Size (Gbp)[a]	Citation for Genome Size[b]	S-phase Duration	Cell/Tissue	Method for Determining S-phase Duration	Citation[b]
Bos taurus (domestic cow)	2.9	Zimin *et al*., 2009	7-9 h	1st cell cycle ova	Double labeling assay	Laurincik *et al*., 1994
Homo sapien (human)	2.9	Collins *et al*., 2004	9.5-10 h	Epidermis	Double labeling assay	Berlingin *et al*., 1992
			≥4 h	Tripronuclear zygotes	3H-thymidine labeling/autoradiographic microscopy	Balakier *et al*., 1993
			3-5 h	Zygotes	3H-thymidine labeling/autoradiographic microscopy	Balakier *et al*., 1993
			5 h	HeLa cell line	PLM	Painter and Drew, 1959
			10-12 h	HeLa cell line	Synchronization and dTTP labeling	Jackson, 1995
			8 h	hESC, somatic IMR90 lines	BrdU labeling, colcemid synchronization/FM	Becker *et al*., 2006

[a]Genome size estimations from sequencing data. [b]Citation references listed below. FCM=flow cytometry; FM=fluorescent microscopy; HU=hydroxyurea; PLM= percent labeled mitosis or percent labeled metaphase

References for Supplementary Tables S1 and S2

Abe Y, Takahashi J, Fukuda H, Yoshida K. 1992. Cell kinetic study of murine jejunal crypts during multiple doses per day. Tohoku Journal of Experimental Medicine **168**, 311-315.
Adams MD, Celniker SE, Holt RA*, et al.* 2000. The genome sequence of *Drosophila melanogaster*. Science **287**, 2185-2195.
Balakier H. 1993. Tripronuclear human zygotes: the first cell cycle and subsequent development. Human Reproduction **8**, 1892-1897.
Barlow PW, Macdonald PDM. 1973. Analysis of the mitotic cell cycle in the root meristem of *Zea mays*. Proceedings of the Royal Society of London B **183**, 385-398.
Barthel D, Matthe B, Potten CS, Owen G, Loeffler M. 2000. Proliferation in murine epidermis after minor mechanical stimulation Part 2. Alterations in keratinocyte cell cycle fluxes. Cell Proliferation **33**, 247-259.
Becker KA, Ghule PN, Therrien JA, Lian JB, Stein JL, Van Wijnen AJ, Stein GS. 2006. Self-renewal of human embryonic stem cells is supported by a shortened G1 cell cycle phase. Journal of Cellular Physiology **209**, 883-893.
Bennett MD, Finch RA. 1972. The mitotic cycle time of root meristem cells of *Hordeum vulgare*. Caryologia **25**, 439-444.
Berlingin E, Heenen M, Galand P. 1992. Measurement of S-phase duration in human epidermis using cyclin immunostaining and 3H thymidine pulse labeling. Archives of Dermatological Research **284**, 238-241.
Blumenthal AB, Kriegstein HJ, Hogness DS. 1974. The units of DNA replication in *Drosophila melanogaster* chromosomes. Cold Spring Harbor Symposia on Quantitative Biology **38**, 205-223.
Cameron IL. 1964. Is the duration of DNA synthesis in somatic cells of mammals and birds constant? Journal of Cell Biology **20**, 185-188.
Cameron IL, Greulich RC. 1963. Evidence for an essentially constant duration of DNA synthesis in renewing epithelia of adult mouse. Journal of Cell Biology **18**, 31-40.
Christov K, Swanson SM, Guzman RC, Thordarson G, Jin E, Talamantes F, Nandi S. 1993. Kinetics of mammary epithelial-cell proliferation in pituitary isografted BALB/c mice. Carcinogenesis **14**, 2019-2025.
Collins FS, Lander ES, Rogers J, Waterston RH, Conso IHGS. 2004. Finishing the euchromatic sequence of the human genome. Nature **431**, 931-945.
Davies POL, Rees H. 1975. Mitotic cycles in *Triticum* species. Heredity **35**, 337-345.
Dhondt S, Coppens F, De Winter F, Swarup K, Merks RM, Inze D, Bennett MJ, Beemster GT. 2010. SHORT-ROOT and SCARECROW regulate leaf growth in Arabidopsis by stimulating S-phase progression of the cell cycle. Plant Physiology **154**, 1183-1195.
Dubatolova TD, Dorogova NV, Omel'yanchuk LV, Chang LS. 2011. Duration of the cell cycle phases in mutants for the tumor suppressor *Merlin* in *Drosophila melanogaster*. Russian Journal of Genetics **47**, 404-408.
Evans GM, Rees H. 1971. Mitotic cycles in dicotyledons and monocotyledons. Nature **233**, 350-351.

Evans LS, Van't Hof J. 1975. The age-distribution of cell cycle populations in plant root meristems. Complex tissues. Experimental Cell Research **90**, 401-410.
Farioli-Vecchioli S, Mattera A, Micheli L*, et al.* 2014. Running rescues defective adult neurogenesis by shortening the length of the cell cycle of neural stem and progenitor cells. Stem Cells **32**, 1968-1982.
Francis D, Davies MS, Barlow PW. 2008. A strong nucleotypic effect on the cell cycle regardless of ploidy level. Annals of Botany **101**, 747-757.
Gahan PB, Hurst PR. 1976. Effects of ageing on the cell cycle in *Zea mays*. Annals of Botany **40**, 887-890.
Gibbs RA, Weinstock GM, Metzker ML*, et al.* 2004. Genome sequence of the Brown Norway rat yields insights into mammalian evolution. Nature **428**, 493-521.
Gressens P, Paindaveine B, Hill JM, Evrard P, Brenneman DE. 1998. Vasoactive intestinal peptide shortens both G1 and S phases of neural cell cycle in whole postimplantation cultured mouse embryos. European Journal of Neuroscience **10**, 1734-1742.
Grif VG, Ivanov VB, Machs EM. 2002. Cell cycle and its parameters in flowering plants. Tsitologiia **44**, 936-980.
Hayashi K, Hasegawa J, Matsunaga S. 2013. The boundary of the meristematic and elongation zones in roots: endoreduplication precedes rapid cell expansion. Scientific Reports **3**, 2723.
Hillier LW, Coulson A, Murray JI, Bao ZR, Sulston JE, Waterston RH. 2005. Genomics in *C. elegans:* So many genes, such a little worm. Genome Research **15**, 1651-1660.
Hillier LW, Miller W, Birney E*, et al.* 2004. Sequence and comparative analysis of the chicken genome provide unique perspectives on vertebrate evolution. Nature **432**, 695-716.
Howlett SK, Bolton VN. 1985. Sequence and regulation of morphological and molecular events during the first cell cycle of mouse embryogenesis. Journal of Embryology and Experimental Morphology **87**, 175-206.
Hu Z, Cools T, Kalhorzadeh P, Heyman J, De Veylder L. 2015. Deficiency of the *Arabidopsis* helicase RTEL1 triggers a SOG1-dependent replication checkpoint in response to DNA cross-links. The Plant Cell **27**, 149-161.
Huard JM, Schwob JE. 1995. Cell cycle of globose basal cells in rat olfactory epithelium. Developmental Dynamics **203**, 17-26.
Jackson DA. 1995. S-phase progression in synchronized human cells. Experimental Cell Research **220**, 62-70.
Jaramillo-Lambert A, Ellefson M, Villeneuve AM, Engebrecht J. 2007. Differential timing of S phases, X chromosome replication, and meiotic prophase in the *C. elegans* germ line. Developmental Biology **308**, 206-221.
Kidd AD, Francis D, Bennett MD. 1987. Replicon size, mean rate of DNA replication and the duration of the cell cycle and its component phases in eight monocotyledonous species of contrasting DNA *C* values. Annals of Botany **59**, 603-609.
Kusanagi A. 1966. Rate of DNA replication in the DNA synthetic period of the barley chromosomes. Chromosoma **20**, 125-132.
Laurincik J, Kopecny V, Hyttel P. 1994. Pronucleus development and DNA synthesis in bovine zygotes in vivo. Theriogenology **42**, 1285-1293.

Lee JH, Arumuganathan K, Kaeppler SM, Papa CM, Kaeppler HF. 1996. Cell synchronization and isolation of metaphase chromosomes from maize (*Zea mays* L.) root tips for flow cytometric analysis and sorting. Genome **39**, 697-703.
Macauley A, Werb Z, Mirkes PE. 1993. Characterization of the unusually rapid cell-cycles during rat gastrulation. Development **117**, 873-883.
Menges M, Samland AK, Planchais S, Murray JA. 2006. The D-type cyclin CYCD3;1 is limiting for the G1-to-S-phase transition in *Arabidopsis*. The Plant Cell **18**, 893-906.
Olszewska MJ, Bilecka A, Kuran H, Marciniak K, Jakubinski J. 1990. Dry mass and protein increase during interphase as a possible factor regulating the cell cycle duration. Caryologia **43**, 43-55.
Painter RB, Drew RM. 1959. Studies on deoxyribonucleic acid metabolism in human cancer cell cultures (Hela).1. The temporal relationships of deoxyribonucleic acid synthesis to mitosis and turnover time. Laboratory Investigation **8**, 278-285.
Reddy GPV, Tiarks CY, Pang LZ, Wuu J, Hsieh CC, Quesenberry PJ. 1997. Cell cycle analysis and synchronization of pluripotent hematopoietic progenitor stem cells. Blood **90**, 2293-2299.
Richard C, Granier C, Inze D, De Veylder L. 2001. Analysis of cell division parameters and cell cycle gene expression during the cultivation of *Arabidopsis thaliana* cell suspensions. Journal of Experimental Botany **52**, 1625-1633.
Sherman FG, Quastler H, Wimber DR. 1961. Cell population kinetics in the ear epidermis of mice. Experimental Cell Research **25**, 114-119.
Shermoen AW, McCleland ML, O'Farrell PH. 2010. Developmental control of late replication and S phase length. Current Biology **20**, 2067-2077.
Sladek TL, Jacobberger JW. 1992. Simian virus 40 large T-antigen expression decreases the G1 and increases the G2 + M cell cycle phase durations in exponentially growing cells. Journal of Virology **66**, 1059-1065.
Takahashi T, Bhide PG, Goto T, Miyama S, Caviness VS. 1999. Proliferative behavior of the murine cerebral wall in tissue culture: cell cycle kinetics and checkpoints. Experimental Neurology **156**, 407-417.
Trunova SA, Dubatolova TD, Omel'ianchuk LV. 1998. Determination of the expression phase of chb(V40) gene in the cell cycle of *Drosophila melanogaster*. Ontogenez **29**, 342-346.
Van't Hof J, Kuniyuki A, Bjerknes CA. 1978. The size and number of replicon families of chromosomal DNA of *Arabidopsis thaliana*. Chromosoma **68**, 269-285.
Vanky P, Brockstedt U, Hjerpe A, Wikstrom B. 1998. Kinetic studies on epiphyseal growth cartilage in the normal mouse. Bone **22**, 331-339.
Verma RS. 1980. The duration of G_1, S, G_2, and mitosis at four different temperatures in *Zea mays* L. as measured with ^{3}H-thymidine Cytologia **45**, 327-333.
Verma RS, Lin MS. 1979. The duration of DNA synthetic (S) period in *Zea mays*: a genetic control. Theoretical and Applied Genetics **54**, 277-282.

Wang AS, Phillips RL, Mi CC. 1986. Cell cycle parameters and accumulation of metaphase cells in maize suspension cultures. Plant Science Letters **46**, 53-61.
Waterston RH, Lindblad-Toh K, Birney E*, et al.* 2002. Initial sequencing and comparative analysis of the mouse genome. Nature **420**, 520-562.
Wolfsberg MF. 1964. Cell population kinetics in the epithelium of the forestomach of the mouse. Experimental Cell Research **35**, 119-131.
Zhang RL, Zhang ZG, Lu M, Wang Y, Yang JJ, Chopp M. 2006. Reduction of the cell cycle length by decreasing G1 phase and cell cycle reentry expand neuronal progenitor cells in the subventricular zone of adult rat after stroke. Journal of Cerebral Blood Flow and Metabolism **26**, 857-863.
Zimin AV, Delcher AL, Florea L*, et al.* 2009. A whole-genome assembly of the domestic cow, *Bos taurus*. Genome Biology **10**.
